# Promotion of microglial phagocytosis by tuftsin stimulates remyelination inexperimental autoimmune en- cephalomyelitis

**DOI:** 10.3892/mmr.2020.11042

**Published:** 2020-03-26

**Authors:** Zheng Bao, Jinqi Hao, Yuhong Li, Fumin Feng

Mol Med Rep 20: 5190-5196, 2019; DOI: 10.3892/mmr.2019.10788

Following the publication of this article, the authors have realized that an error was made with the description of the first and fourth listed affiliation addresses: “North China University of Science and of Technology”, should have been written as “North China University of Science and Technology”. This error also affected the Correspondence box information. Therefore, the author affiliations and addresses, and the Corresponding author information, in this paper should have appeared as follows:

ZHENG BAO^1,2^, JINQI HAO^3^, YUHONG LI^1^ and FUMIN FENG^1,4^

^1^School of Public Health, North China University of Science and Technology, Tangshan, Hebei 063210; ^2^Child Health Division, Tongzhou Maternal and Child Health Hospital of Beijing, Beijing 101101; ^3^School of Public Health, BaoTou Medical College, Baotou, Neimenggu 014040; ^4^Center-Laboratory, North China University of Science and Technology, Tangshan, Hebei 063210, P.R. China

Correspondence to: Dr Fumin Feng, Center-Laboratory, North China University of Science and Technology, 21 Bohai Road, Tangshan, Hebei 063210, P.R. China

E-mail: hblgffm@126.com

The authors regret this error in the presentation of these addresses, and apologize for any inconvenience caused.

